# Mobile phone apps for clinical decision support in pregnancy: a scoping review

**DOI:** 10.1186/s12911-019-0954-1

**Published:** 2019-11-12

**Authors:** Jenny Carter, Jane Sandall, Andrew H. Shennan, Rachel M. Tribe

**Affiliations:** Department of Women and Children’s Health, School of Life Course Sciences, Faculty of Life Sciences and Medicine, King’s College London, London, UK

**Keywords:** Mobile applications, mHealth, Digital healthcare, Decision support systems, clinical, Decision support techniques

## Abstract

**Background:**

The use of digital technology in healthcare has been found to be useful for data collection, provision of health information and communications. Despite increasing use of medical mobile phone applications (apps), by both clinicians and patients, there appears to be a paucity of peer-reviewed publications evaluating their use, particularly in pregnancy. This scoping review explored the use of mobile phone apps for clinical decision support in pregnancy. Specific objectives were to: 1. determine the current landscape of mobile phone app use for clinical decision support in pregnancy; 2. identify perceived benefits and potential hazards of use and 3. identify facilitators and barriers to implementation of these apps into clinical practice.

**Methods:**

Papers eligible for inclusion were primary research or reports on the development and evaluation of apps for use by clinicians for decision support in pregnancy, published in peer-reviewed journals. Research databases included Medline, Embase, PsychoInfo, the Cochrane Database of Systematic Reviews and the online digital health journals JMIR mHealth and uHealth. Charting and thematic analysis was undertaken using NVivo qualitative data management software and the Framework approach.

**Results:**

After screening for eligibility, 13 papers were identified, mainly reporting early stage development of the mobile app, and feasibility or acceptability studies designed to inform further development. Thematic analysis revealed four main themes across the included papers: 1. acceptability and satisfaction; 2. ease of use and portability; 3. multi-functionality and 4. the importance of user involvement in development and evaluation.

**Conclusions:**

This review highlights the benefits of mobile apps for clinical decision support in pregnancy and potential barriers to implementation, but reveals a lack of rigorous reporting of evaluation of their use and data security. This situation may change, however, following the issue of FDA and MHRA guidelines and implementation of UK government and other international strategies. Overall, the findings suggest that ease of use, portability and multi-functionality make mobile apps for clinical decision support in pregnancy useful and acceptable tools for clinicians.

## Background

The use of digital technology in healthcare has been recent and rapid and the advantages of mHealth, i.e. digital health technologies that utilize mobile phones, is seen as a natural progression [1]. Recent UK Government policy recognises value of digital health technology and encourages its integration [2]. Mobile technology has been found to be useful for data collection, provision of health information and communications, particularly in lower and middle income countries, where mobile phones are very common [3–5]. An increasing body of evidence suggests mHealth interventions can improve outcomes and health service utilization [5–7]. One of the particular advantages of mobile phone applications (apps) is that they can be updated regularly, ensuring information is based on current evidence, and they are so readily accessible [8].

Mobile phone health apps are widely used by clinicians as well as patients. A survey of UK medical students (*n* = 257) and junior doctors (*n* = 131) carried out in 2011 found a high level of smart phone ownership (79%, 203/257 and 75%, 98/131, respectively) and mobile app usage (76%, 155/203 and 72%, 71/98, respectively) with both groups expressing an interest in the development of additional apps to enhance their education and professional practice [9]. As technology has moved on in recent years, this is likely to have increased. A more recent survey of 197 Californian obstetrics and gynaecology doctors found that 95% used mobile apps in the clinical setting [10].

There are concerns, however, that without official validation and regulation some medical apps may produce erroneous results and lead to incorrect, inappropriate or even dangerous decisions [11]. In 2015, in recognition of the growing number of medical apps in use, the USA’s regulatory body the Food and Drugs Administration (FDA) issued guidance [12]. This guidance stipulates that if a mobile app is defined as a medical device it will be regulated in the same way as other medical devices. In the UK, the Medicines and Healthcare products Regulatory Agency (MHRA) also considers and regulates medical mobile apps providing they meet the regulatory body’s definition of a medical device [13].

Despite an increasing use of medical mobile phone apps in healthcare, by both clinicians and patients, there appears to be a paucity of peer-reviewed professional journal publications evaluating their use [14]. We decided to undertake a scoping review in order to identify papers providing insights that could inform development of mobile apps for clinical decision support in pregnancy. Specific objectives were to: 1. determine the current landscape of mobile phone apps use for decision support or risk assessment by clinicians in pregnancy care; 2. identify perceived benefits and potential hazards of use in clinical practice and 3. identify facilitators and barriers to implementation of these apps into clinical practice.

## Methods

**Fig. 1:**
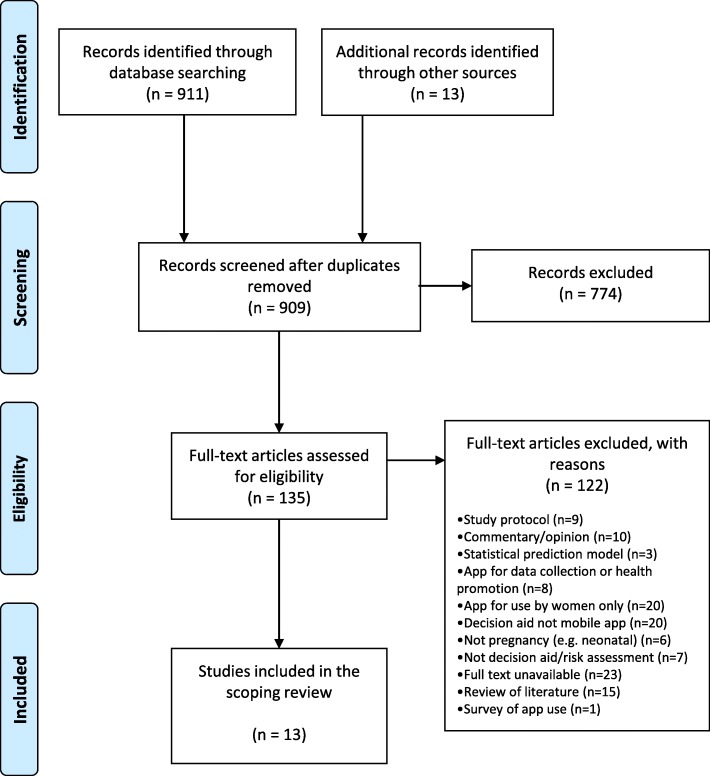
PRISMA 2009 Flow Diagram of results of literature search

Inclusion and exclusion criteria were decided upon prior to initiating a database search and are listed in Table 1. We chose to focus on clinical decision support tools delivered through mobile phone apps, as opposed to other means e.g. clinical guidelines and decision trees. We also wanted to explore those used by clinicians, and therefore excluded apps used solely by pregnant women. The research databases used in the search included: Medline, Embase, PsychoInfo and the Cochrane Database of Systematic Review, with search terms and limits used for each database listed in Table 2. Reference lists and citing articles were also reviewed for other potentially relevant papers. In addition to these research databases, the online journals JMIR mHealth and uHealth, which have a specific focus on digital health, were also searched for papers reporting on pregnancy, labour or birth. After removal of duplicates, the database and JMIR journals search produced a total of 909 articles for screening. Review of the titles and abstracts identified 774 of these to be ineligible based on the inclusion criteria, leaving 135 papers for full text review. Of these, only 13 were eligible for inclusion, with 122 being excluded for the reasons shown in the PRISMA flow diagram (Fig. 1).

**Table 1:** Inclusion and exclusion criteria for the scoping review

Inclusion	Exclusion
Mobile phone applications (apps) for decision support or risk assessment in pregnancy	Decision aids not utilizing mobile app technology, e.g. clinical guidelines/models/decision trees Apps for data collection or delivery of information/health promotion Statistical prediction models
Primary research or report of app development and evaluation published in peer reviewed journals	Literature review Study protocols Commentaries or editorials
App for use by clinicians or both clinicians and pregnant women	App for use by pregnant women only

**Table 2:** Search terms and limits for the scoping review

	Search term		Search term	Limit
Medline (*n* = 598)	Pregnancy OR Exp Labour, Obstetric OR Labour OR Premature Birth OR Obstetric Labor, Premature OR preterm.mp	AND	mHealth.mp OR mobile application.mp OR Exp Mobile Applications OR smart phone.mp OR Exp Smartphone OR Decision aid$.mp OR Risk assessment tool$.mp OR Predictive model.mp OR App.mp	Papers published between 2007 (when the iPhone and first mobile apps were available) and June 2018; Humans
Embase (*n* = 187)	Pregnancy OR Exp Labour, Obstetric OR Labour OR Premature Birth OR Obstetric Labor, Premature OR preterm.mp	AND	mHealth.mp OR mobile application.mp OR Exp Mobile Applications OR smart phone.mp OR Exp Smartphone OR Decision aid$.mp OR Risk assessment tool$.mp OR Predictive model.mp OR App.mp	Papers published between 2007 (when the iPhone and first mobile apps were available) and June 2018; Humans; Full text (as large number, *n* = 479, of abstract only references were returned)
PsychInfo (*n* = 61)	Pregnancy OR Exp Labour, Obstetric OR Labour OR Premature Birth OR Obstetric Labor, Premature OR preterm.mp	AND	mHealth.mp OR mobile application.mp OR Exp Mobile Applications OR smart phone.mp OR Exp Smartphone OR Decision aid$.mp OR Risk assessment tool$.mp OR Predictive model.mp OR App.mp	Papers published between 2007 (when the iPhone and first mobile apps were available) and June 2018; Humans
Cochrane Database of Systematic Reviews (*n* = 46)	Pregnancy: tl, ab, kw (including word variations)	AND	mHealth OR decision aid OR risk assessment tool OR smart phone OR mobile phone	No limits
JMIR mHealth and uHealth (*n* = 43)	Pregnancy OR Labour OR Labor OR Birth	AND	Risk OR Decision	No limits

*Exp* explode, *m.p* keyword search, *$* wildcard symbol, *tl* title, *ab* abstract, *kw* keyword

A formal review of quality was not undertaken because this was not a systematic review with the aim of establishing the efficacy of an intervention. Charting and thematic analysis was carried out using NVivo qualitative data management software (version. 11) and Framework Analysis [15]. Characteristics of the papers and the apps they describe are shown in Table 3. These include a description of the report or study design, setting, participants and time period, area of pregnancy focus and app characteristics. The main findings and conclusions reported by the authors were explored using thematic analysis.

**Table 3:** Characteristics of papers and mobile applications reported in included papers

Reference	Description of paper and study design	Setting, participants and time period	Area of focus in pregnancy	App functions				App characteristics
				Decision support	Data collection	Communi-cation	Connect-ed device	
Watson et al. (2017) [16]	Study assessing the reliability of risk prediction scores incorporating in the app. Comparison of the QUIPP predicted risk within 7 days to the actual delivery rates. Also investigated the impact of using the QUIPP app relative to a treat-all strategy at 24–29 + 6 weeks (as per NICE Preterm Labour guideline 2015).	UK. 355 women with threatened preterm labour between 2010 and 2015.	Preterm birth	Yes	No	No	No	Risk of preterm delivery in symptomatic women calculated using risk factors and test results.
Dunsmuir et al. (2014) [17]	Describes development of app and results of usability study. This paper describes the development process, including challenges and solutions, of the PIERS on the Move (POTM) is a low cost, easy-to-use, mobile health (mHealth) application for accurately predicting the risk of adverse outcomes associated with pre-eclampsia in pregnant women.	Cape Town, South Africa. 202 women had been assessed with the POTM application. A total of 37 nurses and midwives evaluated the user interface through three usability studies. November 2011 to January 2013.	Pre-eclampsia	Yes	Yes	No	No	App calculated a risk score using clinical findings (including measurements by pulse oximeter connected to a smartphone). Based on the risk score, the application provided recommendations on treatment, referral, and reassessment.
Jeon et al. (2016) [18]	Describes development of app and results of evaluation study. Paper reports the development and evaluation of four mobile applications that provide tailored nursing recommendations for metabolic syndrome management in pregnancy. Evaluation included the algorithm proficiency and efficiency, user interface, usability, and effectiveness. Usability evaluated using different tools for each condition.	Setting and time period not explicit (corresponding author based in Korea). Paper reports “evaluations by experts and users.” But does not specify any detail or numbers involved.	Metabolic syndrome in pregnancy, including, obesity, diabetes, hypertension, and hyperlipidaemia.	Yes	No	No	No	Authors report: “Mobile applications provide tailored nursing recommendations for metabolic syndrome management, e.g. “when a patient intakes more calories than needed, the alert function can alert the patient by sending a message based on the daily calorie intake that the diary function has helped the patient to track. p. 512” No further detail or examples reported.
Jonas et al. (2016) [19]	Describes development of app and results of evaluation study. Paper describes the development and evaluation of a smartphone-based imaging and automated analytical tool which incorporates the Congo Red Dot (CRD) test. This test assesses the presence of misfolded proteins in urine, and shows promise as a diagnostic and prognostic tool for preeclampsia. Stage 1: evaluation of a preliminary version of image processing software tool using stored images. Stage 2: testing improvements in real-time on newly prepared standardized CRD arrays and analysed the results for agreement. Stage 3: Analysis of test results across four operators, including untrained personnel (*n* = 1) who did not receive any instruction or prior knowledge of the system.	Setting and time period not explicit. Corresponding author based in Germany. No patient participants.	Pre-eclampsia	Yes	No	No	Yes	Smartphone application guides the user through seven easy steps that can be used by non-specialized personnel, through test image acquisition to interpretation of result.
Lim et al. (2015) [20]	Describes development of app and results of 2 usability and feasibility studies. Paper reports findings of study assessing the usability and feasibility of PIERS on the Move, an App for pre-eclampsia triage, with mid-level health workers, for iteratively refining the system. Two usability studies were performed with the potential end-users. Each step in the development process used the findings of the previous, thus improving on the design and features available in the app.	Usability study 1: evaluation by advanced midwifery students at Tygerberg Hospital (Cape Town, South Africa), (*n* = 15). Usability study 2: evaluation of the next iteration by maternal nursing staff at Frere Maternity Hospital (East London, South Africa), (*n* = 22). November 2012 to December 2013.	Pre-eclampsia	Yes	Yes	No	Yes	“Pre-eclampsia Integrated Estimate of RiSk (PIERS) on the Move (PotM) is a low cost, easy-to-use, mobile health (mHealth) platform that has been created to aid health workers in making decisions around the management of hypertensive pregnant women. The app combines two previously successful innovations into a mHealth app: the miniPIERS risk assessment model and the Phone Oximeter.”
Mackillop et al. (2014) [21]	Describes development of app and results of evaluation study. Paper describes development of a prototype software application for the management of women with or at high risk of Gestational Diabetes. A custom website was built for clinician review of the data transmitted by the smartphone. After system refinement, further evaluation was undertaken for usability and reliability in a 48-patient service development project.	UK. Seven women participated in the first testing phase and 50 of the 104 women approached volunteered to test the system in the service development phase.	Gestational Diabetes	Yes	Yes	Yes	Yes	Authors state that the: “Functional objectives included the ability to: 1. Allow women to accurately and easily record blood glucose measurements, which are then automatically uploaded to a website. 2. Allow health care professionals to access these measurements remotely and respond quickly to them, thus potentially improving glycaemic control without the need for more intensive face-to-face contact. 3. Allow 2-way communication between women and health care professionals. 4. Promote user participation (empowerment) of pregnant women in their medical management.”
Marko et al. (2016) [22]	Prospective observational study assessing feasibility, efficacy and satisfaction. Paper reports findings of a prospective observational pilot study to determine the feasibility of monitoring patients remotely in prenatal care using a mobile phone app and connected digital devices. As measures of the feasibility of the system, participants were studied for engagement with the app, accuracy of remote data, efficacy of alert system, and patient satisfaction. Patient satisfaction was measured using a 12-question survey that was completed by participants after 20 weeks of platform usage.	Department of Obstetrics & Gynecology at the George Washington University Hospital, USA. *n* = 8 women with low risk pregnancy in the first trimester. July 2014 to January 2015.	Weight and blood pressure monitoring in pregnancy	Yes	Yes	Yes	Yes	Mobile phone app with a connected digital weight scale and blood pressure cuff for at-home data collection for the duration of pregnancy. At-home data was assessed for abnormal values of blood pressure or weight to generate clinical alerts to the patient and provider.
Peleg et al. (2017) [23]	Mixed methods study of compliance, satisfaction and quality of life. The MobiGuide project aimed to establish a user-friendly, patient-centred mobile decision-support system for patients and for their care providers, based on the continuous application of clinical guidelines and on semantically integrated electronic health records. The objective of this paper was to evaluate whether the initial deployment of the MobiGuide system, for two different clinical domains - atrial fibrillation (AF) and gestational diabetes (GDM) - had achieved three main outcomes: (a) high patients’ and care providers’ compliance to clinical-guideline based monitoring reminders and recommendations, (b) high patients and care providers’ satisfaction, and (c) increased patients’ quality of life.	Italy and Spain. The study involved ten AF patients from IRCCS Foundation “Salvatore Maugeri”, Pavia, Italy and twenty GDM patients from Parc Tauli Sabadell University Hospital, Sabadell, Spain. As a control group for GDM, researchers referred to data from a historical group of 247 patients, similar in characteristics, who had been followed up during 2010–2013 at the same GDM clinic. April to December 2015.	Gestational diabetes	Yes	Yes	Yes	Yes	Authors report: “MobiGuide is a remote chronic-patient management system that has five main objectives: (1) Increasing patient safety and quality of care through provision of personalized ubiquitous decision-support to the patients. (2) Semantic data integration into a personal health records. (3) Creation of a generic architecture that supports interoperation with a variety of portable sensors, and different hospital electronic health records. (4) Distribution of the decision support system (DSS), between a mobile DSS that runs on the patient’s smart phone and a backend DSS that is accessible via the Internet by the patients’ care providers. (5) Performance of intelligent data analysis, to discover clinical data patterns in individual patients, thus providing additional decision-support.” In the GDM domain, blood glucose monitor and sphygmo-manometer were connected to the patient’s smart phone by Bluetooth.
Stroux et al. (2016) [24]	Mixed methods study of feasibility and acceptability. Paper describes findings of a mixed methods feasibility study to evaluate a smart phone based system designed to identify fetal compromise. The feasibility assessment was designed to evaluate whether frontline healthcare workers could operate the study equipment (1D foetal Doppler, pulse oximeter and recording application) and record signals successfully using a smart phone. The study also set out to assess user need and to assess the acceptability by both healthcare provider and patient.	Guatemala. *n* = 22 pregnant women. Written feedback was provided by 6 members of staff.	Fetal compromise	Yes	Yes	Yes	Yes	A smartphone-based system including peripheral sensors, pulse oximeter and handheld Doppler for the identification of foetal compromise. Designed for use by illiterate birth attendants, the system uses pictograms, audio guidance, local and cloud processing, SMS alerts and voice calling.
Tsai et al. (2014) [25]	Paper describes findings of a feasibility study aimed at determining the extent to which community health workers could be trained to conduct case finding using short and ultrashort screening instruments programmed into mobile phones. Pregnant women were recruited independently in two cross-sectional studies and assessed for antenatal depression.	Khayelitsha, South Africa. May 2009 to September 2010 (*n* = 1144) and May 2010 to February 2011. (*n* = 361).	Antenatal depression	Yes	Yes	No	No	In both studies, the Xhosa version of the EPDS-10 was administered using survey software programmed into a mobile phone.
von Dadelszen et al. (2015) [26]	Paper describing observations noted during development of app. This paper describes observations noted during development of the PIERS (Pre-eclampsia Integrated Estimate of RiSk) models that identify pregnant women with pre-eclampsia who are most likely to develop life-threatening complications, and suggests recommendations for development of mHealth in perinatal care. The authors had developed and validated two outcome prediction models, the PIERS (full and mini). Both models have accurate ability to identify women at low risk of developing imminent complications.	For use in low and middle income countries. 2011.	Pre-eclampsia and other potentially life threatening conditions.	Yes	Yes	Yes	Yes	Authors state: “The PIERS on the Move (POM) smart phone app integrates miniPIERS and clinical decision algorithms to support community health care professionals (cHCPs) as they provide prenatal care, diagnose pre-eclampsia, and initiate lifesaving therapies in the woman’s home prior to urgent transfer to an effective facility. The researchers have also developed a modified blood pressure device (Microlife 3AS1–2; Microlife, Widnau, Switzerland) specifically for use in low- and middle-income countries (LMICs), which fulfils WHO requirements for suitability for use in low-resource settings. A traffic light early warning system has been incorporated into the device, to alert users to abnormalities in blood pressure and pulse, using these developed shock index thresholds along with well-recognized thresholds to indicate hypertension in pregnancy.”
Battle et al. (2015) [27]	Mixed methods evaluation of program using app. Report of findings from a mixed methods study qualitative and quantitative data evaluation the “mHealth for Safer Deliveries” program - an integrated mobile health intervention on maternal care utilization. The program was designed to address each of the “three delays” to receiving skilled care at delivery: (1) the decision to seek care; (2) reaching skilled care; and (3) the provision of adequate care once at the health facility.	Zanzibar, Africa. January 2013 to December 2014. Qualitative interviews -September-October 2014 in all districts using semi-structured interviews (women, *n* = 27; community health workers, *n* = 25; health facility workers, *n* = 12). Quantitative data were collected January 2013 and December 2014 (*n* = 13,231).	To encourage facility birth	Yes	Yes	Yes	No	The program supported community health workers trained to use a phone with a user-friendly decision-support application. This enabled them to: 1. Counsel the mother and family on healthy behaviours and recognizing danger signs; 2. Record permissions from husband and family members agreeing to a facility-based delivery; 3. Screen women (and their babies) for complications from pregnancy up to a week after delivery and refer them as needed to the health facility; 4. Use mobile banking to pay for transportation to the health facility when the woman is referred, paying for transport without ever touching cash; 5. Use text or voice communication to notify a health facility that a woman is in transit.
Vélez et al. (2014) [28]	Mixed methods study evaluating a program using app. The Millennium Villages Project (MVP) was an integrated rural development program to achieve the Millennium Development Goals (MDGs) in low-income rural Africa by 2015. The Millennium Village Health System (MVHS) was a major component of the project, whose core strategy was to ensure universal access to services free of charge at the point of care, with a continuum of services from the household to the clinic and the referral hospital. This paper describes a descriptive usability study composed of 3 phases to evaluate an mClinic prototype: 1) hybrid lab-live software evaluation of mClinic to identify usability issues; 2) completion of a usability questionnaire; and 3) interviews that included low-fidelity prototyping of new functionality proposed by midwives.	Bonsaaso, Ghana. All midwives working in the cluster of MVP (*n* = 7). May 2011.	Access to maternity care.	Yes	Yes	Yes	No	A mobile health (mHealth) application, known as mClinic, captures data for managing patient care, program evaluation and monitoring, decision making, and management, and allows midwives to access the MVG-Net.

## Results

### Characteristics of the papers included in the review

Details of the 13 included papers are shown in Table 3. Of the included papers, one [16] reported the reliability of a clinical decision support tool for calculating risk of preterm birth. The majority (*n* = 10) described early development of the mobile app with results of feasibility, usability studies and/or satisfaction studies [17–26]. Two reported results of studies evaluating maternity care projects in which the app was a central component of care delivery [27, 28].

Seven papers reported on studies or projects based in low and middle income countries, including Africa and Guatemala [17, 20, 24–28]. Two were based in the UK [16, 21], one in Spain and Italy [23] and one in USA [22]. In two papers the location of the project was unclear, however one of the corresponding authors was based in Korea [18] and the other in Germany [19]. All were published between 2014 and 2017: four in 2014; three in 2015; four in 2016 and two in 2017.

Four papers reported on apps focusing on preeclampsia [17, 19, 20, 26]. Three of these, however, all referred to the same project, Pre-eclampsia Integrated Estimate of Risk (PIERS) [17, 20, 26]. Gestational diabetes was the focus for two papers [21, 23]. The aims of the maternity care projects were to increase the number of births in a health facility in Zanzibar [27] and to improve access to maternity care for women in Ghana [28]. The pregnancy focus of each of the remaining five papers were: metabolic syndrome [18]; weight and blood pressure monitoring [22]; identification of fetal compromise [24]; antenatal depression [25] and preterm birth [16].

### Thematic analysis of the main findings and conclusions as reported by the authors

#### Theme 1: acceptability and satisfaction

All papers reporting on acceptability, feasibility, usability and/or satisfaction were generally positive, both with the mobile app being evaluated, and also with the care it was designed to support. This was demonstrated by direct questioning and evaluation tools, but also by increased patient engagement with, for example, compliance with self-monitoring [7, 21, 23]. Increased confidence of health providers, enhanced positive relationships and trust in the professionals and feelings of support and safety were also reported [18, 21, 22, 27]. Validation of data and monitoring readings were often a feature of the app, and this was recognised by clinicians as a valuable improvement in care [17, 24]. Additionally, apps appeared to help clinicians identify priorities and they recognized the potential for the system to be time saving. The automatic transfer of data to electronic central databases or health records was also identified as a useful mechanism which could save clinicians’ time as they could remotely review the data in advance of the patient’s hospital appointment [23, 28]. Alerts systems were utilised in some apps to remind patients of, for example, appointments, medication, and monitoring [21, 22], or alert remote clinicians who could respond with advice, either directly to the patient or their local care givers [21, 24].

#### Theme 2: ease of use and portability

Most medical app users were familiar with smart phones, and the benefit of portability was regarded as a great asset [22, 27]. Some users reported problems which were often related to the phone’s features, e.g. difficulties with entering data on a small mobile phone screen and the need for scrolling [17, 20]. Adaptation of features such as reducing the need for scrolling by having fewer data on each form, training and on-phone manuals were used to address these issues in later stages of app development [18, 24].

With the relative low cost of smartphones and convenience in terms of weight and size, along with the increasing connectivity to mobile networks, mobile apps appear to be accepted as an excellent opportunity for improving healthcare, particularly for those in low resource settings. One reason, proposed by a number of authors of the papers included in this review, is that less educated health care staff can be trained in providing front-line care using devices that are easy to use, with internal validation and warning alerts, with the added benefit of support from remote experts [24–26].

#### Theme 3: multiple functionality

The versatility and multi-functionality of smartphones appeared to be an important issue in the papers reviewed. As decision support tools, mobile apps can utilize statistical prediction models or decision trees and make recommendations for action based on input of individual risk factors and test results [16, 17, 23]. In addition to decision support, however, most apps (10/13) were also used for data collection, communication, or both. Other apps also incorporated Bluetooth internet connectivity with other devices: pulse-oximetry [20, 24]; blood glucose monitors [21, 23]; blood pressure monitors [22, 23]; digital weighing scales [22] and fetal Doppler devices [24]. One mobile app utilized the smartphone’s own camera for processing pictures used in the Congo Red Dot test to assess the presence of misfolded proteins in urine [19]. This test has been proposed as a possible diagnostic test for pre-eclampsia that could be particularly useful in low resource settings where more sophisticated laboratory facilities are unavailable.

Communication between patients and healthcare workers, or between healthcare workers and colleagues or other experts, was valued as an important element in the success of the projects in which the apps played a central role [17, 21, 27]. This appeared to be so whether the communication was carried out directly through the app, or simply by the user being able to communicate using the same device, i.e. mobile phone.

Data collection, validation, transfer and integration with other health records and research databases, and the ability to set alerts, as noted above, along with other integrated features of mobile phone technology, such as time stamping and Global Positioning System (GPS) tracking of phone location, were also noted as important and useful attributes because, for example, the time and place of the clinical visit could be recorded [17, 21, 23, 24, 28].

Delivering healthcare interventions through mobile technology also provided the opportunity to adapt programmes relatively easily to account for specific needs of the end-users. Accessibility was enhanced, e.g. picture and video instructions for illiterate users [24]. Language and cultural diversity issues were also relatively easily addressed and incorporated into different versions of the app [17, 21, 24].

#### Theme 4: the importance of user involvement in development and evaluation

The importance of user involvement in the development and evaluation of their app was emphasized in several papers [17, 20, 26, 28]. The authors noted that this was not only a key step in enhancing the acceptability and usability of the device/programme, but also a mechanism by which they could foster engagement by local stakeholders, community leaders and healthcare funders. This interaction was recognised as part of the pathway to ensure acceptability of the programme and to maximize its chances of being sustained.

## Discussion

This scoping review has identified and considered a number of relatively recent papers, mainly reporting early stage development and feasibility or acceptability studies designed to inform further development of the mobile app the paper was concerned with. The number of papers identified was relatively small compared to the number of medical apps readily available for download onto mobile devices. It is likely that many clinicians and other health care professionals are using them on an ad hoc basis. However, there are still only very few peer-reviewed publications in high quality professional journals that can confirm their utility, reliability, effect on outcomes and successful implementation or scale up. None of the papers reported application for regulatory approval by either the FDA or MHRA.

It is possible that the search strategy employed may have missed some important papers due to the lack of standardised search terms associated with the relatively new field of mobile healthcare. The lack of unified language has been previously identified and efforts to address this made, such as the WHO “Classification of Digital Health Interventions” [29]. However, these are relatively recent, and may take some time to become apparent in the literature.

An extensive number of potentially eligible papers required a review of the full text because the nature of the decision support tool or mobile app was not clear from the title or abstract alone. In addition, the speed with which new papers are published makes efforts to undertake a truly comprehensive review of such a fast growing literature base challenging.

Our objectives were to: 1) determine the current landscape of mobile phone app use for clinical decision support in pregnancy; 2) identify perceived benefits and potential hazards of use and 3) identify facilitators and barriers to implementation of these apps into clinical practice. These objectives have largely been met through thematic analysis. The findings are consistent with the widely used “Theory of Acceptance Model” [30]. This model proposes that two particular beliefs, “perceived usefulness” and “perceived ease of use”, are primarily important in predicting future use of computer software. It is, therefore, not surprising that the apps referred to in this review were generally considered acceptable. The issue of data security, however, briefly mentioned in two papers [17, 28] did not appear to be particularly important. Where it had been raised as a concern, password protection at app, rather than phone, level [28] and data encryption [17] appeared to provide acceptable solutions. This may become a more important issue in the future, however, following recent scandals regarding the misuse of personal online data [31].

This scoping review has considered papers reporting on mobile phone apps for clinical decision support in pregnancy. It appears that the body of literature relating to this precise area remains sparse and relatively recent. No papers were found of studies reporting effects on clinical outcomes, although the two papers on programmes to improve healthcare utilization reported success. It is expected, however, that more publications will follow in due course, as the papers reviewed were largely reporting results of feasibility studies of projects that will have entered later phases of development. The situation is also likely to improve in response to calls for the adoption of suitable monitoring and evaluation frameworks, part of the World Health Organisation’s “Global Strategy on Digital Health 2020-2024”, which is currently open for public consultation (April 2019) [32].

## Conclusion

This review highlights the benefits of mobile apps for clinical decision support in pregnancy and potential barriers to implementation, but reveals a lack of rigorous reporting of evaluation of their use and data security. This situation may change, however, following the issue of FDA and MHRA guidelines and implementation of UK government and other international strategies. Overall, the findings suggest that ease of use, portability and multi-functionality make mobile apps for clinical decision support in pregnancy useful and acceptable tools for clinicians.

## Data Availability

All data generated or analysed during this study are included in this published article.

## Abbreviations

App: Mobile or smartphone application
FDA: Food and Drugs Administration (USA)
MHRA: Medicines and Healthcare products Regulatory Agency (UK)
